# Facial defects reconstruction by titanium mesh bending using 3D printing technology: A report of two cases

**DOI:** 10.1016/j.amsu.2022.103837

**Published:** 2022-05-20

**Authors:** Wafik Mayo, Aya Haji Mohamad, Hani Zazo, Aya Zazo, Mais Alhashemi, Aya Meslmany, Bakr Haddad

**Affiliations:** a Faculty of Medicine, University of Aleppo, Aleppo, Syria; b Mashabek, Aleppo, Syria; c Düsseldorf, Germany; d CME Office, Faculty of Medicine, University of Aleppo, Aleppo, Syria; e Faculty of Mechanical Engineering, Engineering Materials Science, University of Aleppo, Aleppo, Syria; f Maxillofacial Surgery Department, Aleppo University Hospital, Aleppo, Syria

**Keywords:** Facial defect, Titanium mesh, War injury, Polylactic acid, Case report

## Abstract

**Introduction:**

Facial injuries and deformities have received special attention during the previous decades for their functional, esthetic impairment, surgical challenges related to the location of the intervention, and their relationship to a lower survival rate. Moreover, there have been many surgical reconstructive methods due to the different materials and tools available and thus the final results following the surgical intervention.

**Case presentation:**

This study was conducted on two patients with severe war injuries; they both suffered from a significant loss in one or more of the following bones: the zygomatic bone, maxilla, nasal bone, infraorbital rim, and mandible. They were treated using preshaped 3D titanium mesh implants that were made using polylactic acid (PLA) material. The final shape was identified depending on pregenerated multislice 3D modeling using computed tomography (CT) scan.

**Clinical discussion and conclusion:**

The patient-specific titanium implants produced using polylactic acid (PLA) have been an important option for reconstructive surgical interventions in facial injuries. It has achieved a better outcome in comparison with manual bent titanium mesh in terms of anatomical symmetry, overall operating time, functional and esthetic impairment. These points helped achieve better care for both civilian and war injuries associated with bone loss.

## Highlights

• Facial injuries are of significant consideration especially during war time.
• Using titanium plates has increased due to its biocompatibility and rigid fixation.
• A printing model for the skull using polylactic acid is a successful approach.
• Pre-shaped mesh plates reveal better surgical outcomes.

## 1 Introduction

The most commonly affected bones in facial trauma are orbital walls and floor, which may lead to several complications such as diplopia, visual acuity disturbance, enophthalmos, and hypogeous [1].

Using a three-dimensional reconstructive implant can avoid many complications [2]. Recently, combining 3D imaging and a titanium mesh and implanting the mesh in the damaged orbit place is giving effective results [3]. In the last 45 years, alloplastic methods have been used for tiny damages; in addition, they have a lot of benefits, for example, tinny, bio-compatibility, solid and bright, radio-opaque without creating artifacts in radiographic investigations [4]. In addition, titanium implantation is helpful established in several facial operations containing facial bone reformation [4].

This case was prospectively reported two patients suffered from a war injury that caused significant bone loss in the face in Aleppo, Syria, and managed in a low-expensive procedure.

## 2 Case presentation

**Fig. (1):**
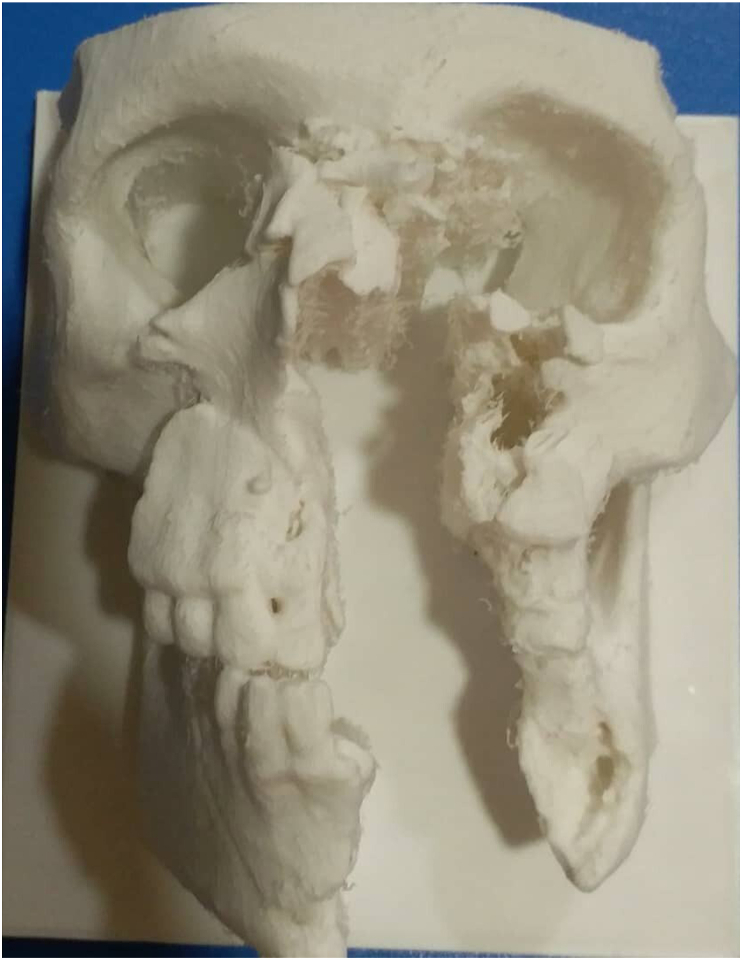
3D reconstruction based on CT scan slices in the first case.

A 19-year-old male was referred to our institution in 2021. He suffered from a severe gunshot wound injury (GSW) that caused significant loss in the zygomatic bone, maxilla, nasal bone, infraorbital rim, and mandible with extensive wounds in the skin and the soft tissue. Initially, soft tissue interventions were performed in order to stop bleeding and sew the wounds. A few months later, when the general condition had improved, he underwent computed tomography (CT) scan to generate multislice 3D modeling of both affected and unaffected face bones (Fig. 1).

The other case was an 18-year-old female who was referred to our institution for evaluation and treatment plan 8 years after a war injury to the left side of the face, including left eye loss and significant loss in the lower-left edge of orbit in 2013. She has also undergone a CT scan to generate slices for the 3D modeling template.

Regarding both cases, the software package processed the CT slices to provide a mirrored 3D shape of the skull, identical to that of the injured young man, including the affected and the unaffected bones. Our simple 3D printer (Ender 3 Pro 3D printer, China) uses polylactic acid (PLA) materials for producing 3D printed objects which have excellent biocompatibility. This allows us to safely adapt and pre-bend the reconstruction titanium mesh on the produced PLA skull prior to surgery to ensure symmetry and less surgical time.

**Fig. (2):**
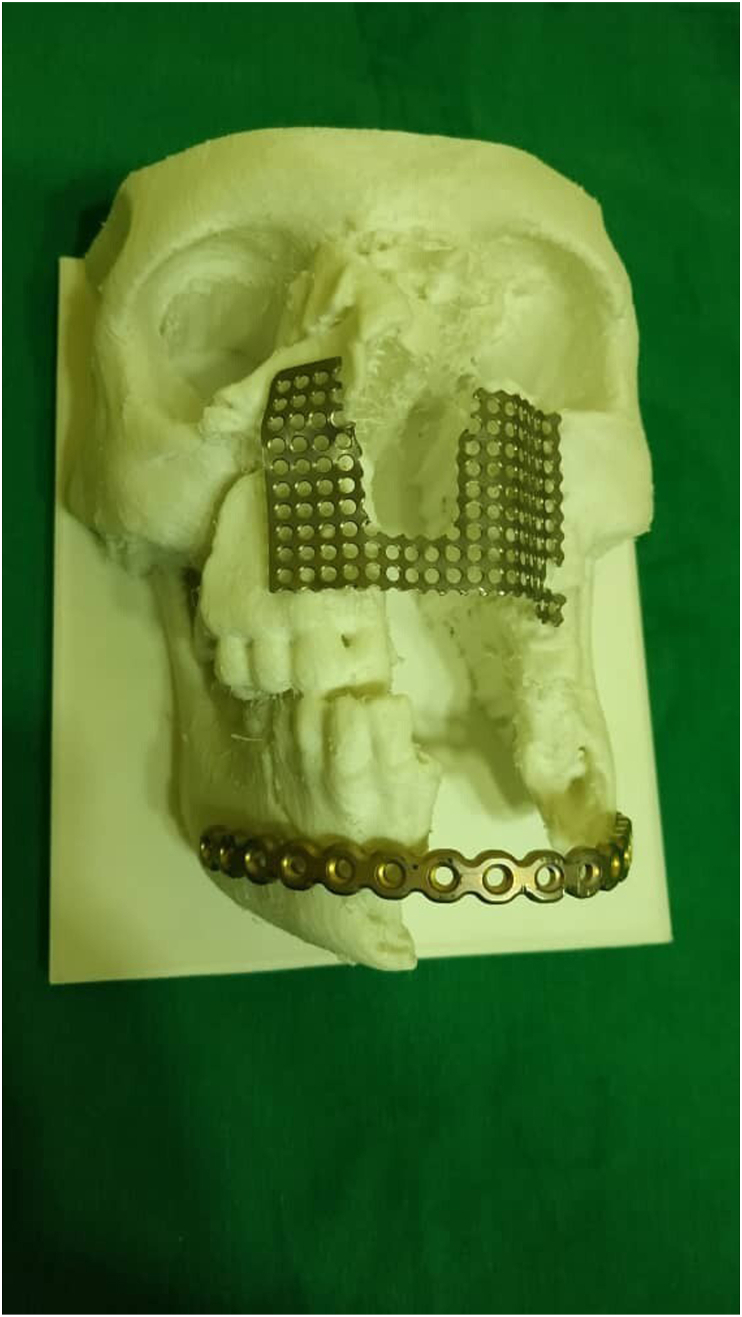
Molding titanium mesh according to 3D printed template in the first case.

**Fig. (3):**
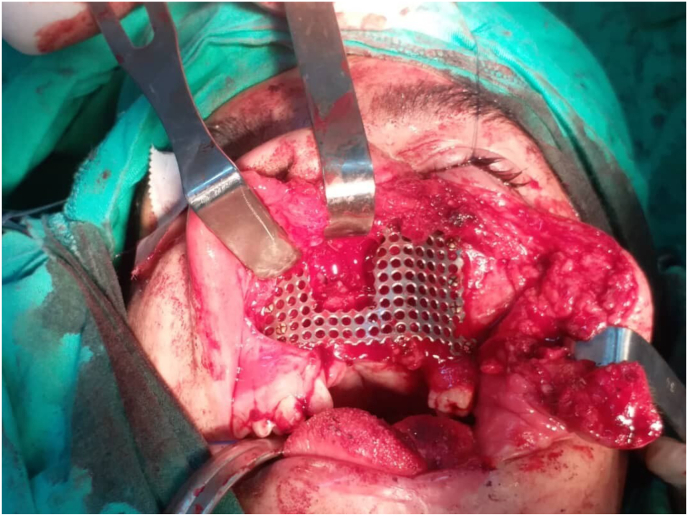
Postoperative aspect showing the reconstructed area in the first case.

The titanium mesh was generated with a thickness of 0.5 mm based on the shape of the 3D printed skull of the affected area (Fig. 2). The intervention on the area was using the Weber Ferguson surgical approach, which includes an incision under the lower eyelid extending to an incision on the side of the nose, then an incision around the nasal wing, finally an incision on the middle line of the upper lip. Then, after sterilizing the mesh, the patient's specific manufactured titanium was implanted and fixed using mini-screws made of titanium as well (Fig. 3).

In both cases, implants' position was assessed using postoperative CT scans. The same type and thickness of the mesh were used in both cases as well.

Regarding the time for each procedure, the 3D skull printing lasted about 45 minutes for each case. The time of bending the titanium mesh was 2.5 hours in the first case and 2 hours in the second. The surgical intervention for mesh implantation took less than 2 h in both cases.

This case report has been reported in line with the SCARE 2020 Criteria [5].

## 3 Discussion

Craniomaxillofacial deformities (CM) increase in number during war times [6], and they require precise reconstruction, as they affect not only the main functions like vision, swallowing, speech, breathing, and mastication; but also the physical appearance [7].

The use of digital surgical technologies has developed in CM reconstructive surgeries, as they enhance surgical procedures and lead to better outcomes for restoring both form and function [8].

The combination between three-dimensional (3D) techniques and radiology allowed the surgeon to better understand the anatomical surgical location of the patient and its exact pathology because every patient is a unique case that requires its own understanding. However, these (3D) images were still displayed in two dimensions, and there was a need to replicate the exact anatomical structure through a process called biomodelling. In 1990, Mankovich et al. reported the 1st bio model through rapid prototyping (RP) technology [9].

The optimal correction method of CMF deformities aims to achieve the ideal normal anatomical reconstruction of the defect. Numerous materials, including autologous such as bone graft, and allogenic, and alloplastic such as titanium mesh, have been widely described, and each material has its advantages and disadvantages [10]. Titanium composites play an important role in the medical field due its strength, lightness and resistance to corrosion. Because of its low iron component, titanium does not produce significant artifact on radiological imaging. Finally, it forms a fibrous tissue between the implant and the bone [11].

These two surgeries aimed to reconstruct large areas of bone loss, including zygomatic, mandibular, maxillary, nasal, and orbital bones. This reconstruction could not be achieved by using autologous materials due to the large bone loss and the associated disadvantages of donor site morbidity and the difficulty in contouring, or by using allogenic materials because of the disadvantage of viral infections [10].

Therefore, there has been an increasing shift to the use of titanium plates in various CMF defects due to its biocompatibility, availability, easy contouring, and rigid fixation [12].

Titanium mesh and plates can be applied in four different ways. First, it can be formed intraoperatively. Second, it can be preformed using a plastic skull. Third, a 3D preformed mesh can be purchased off the shelf. Fourth, it can be custom-made using stereolithography [13].

Raisia et al. found in their study that using custom-made implants in the reconstruction of orbital floor fracture led to better results when compared to intraoperative manual bending [14].

In this case, we performed reconstructive surgery in two patients using pre-bent titanium mesh plates on a 3D skull model. The implant was bent and formed on a 3D printed model using cloning technology which demonstrated the injured bones. Even without mirroring the unaffected side, we were able to cover the area of defect. This approach was cost-effective and used efficient material during wartime in Syria.

Similar studies reported the reconstruction of maxillary and mandibular deformities using a custom-made titanium mesh. This process requires titanium powder [15]. Due to the unavailability of the titanium powder in wartime and the expensive tools of this approach, we formed the implants preoperatively on a 3D skull model using a simple 3D printer. The 3D model clearly demonstrated the affected and non-affected bones and enabled the implant formation on them.

Cui et al. discussed in their study the application of pre-shaped titanium implants on a 3D skull model in the CMF reconstructive surgery [16]. However, their model was made from resin material. In our study, the rapid prototyping skull model was manufactured using polylactic acid (PLA) material, which is made from sustainable resources and is more cost-effective, and serves the same promising outcome [17].

As a result of this approach, facial symmetry was achieved using advanced digital technologies but also cost-effective materials. Pre-shaped mesh plated shortened the surgical duration due to complete implant preparation preoperatively and did not require great adjustments in comparison with the intraoperative approach. Postoperative trauma was reduced in comparison with the conventional approach. Finally, the surgical team's effort was saved due to prior preparation.

## 4 Conclusion

The three-dimensional printing technique, using polylactic acid (PLA) for preparing titanium mesh, has shown better outcomes in restoring the bone structure and maintaining function. As many areas in the world cannot provide titanium powder for preoperation modeling, this technique could be cost-effective as well as time and effort-saving technology in dealing with bone loss injuries and craniomaxillofacial deformities.

## Ethical approval

Writing case repots does not require ethical approval in University hospitals in our country.

## Funding resources

There is no source of funding for this paper.

## Author contribution

Wafik Mayo: data collection, wrote the manuscript, revision. Aya Haji Mohamad: wrote the manuscript, revision. Hani Zazo: wrote the manuscript, revision. Aya Zazo: data collection, critical revision. Mais Alhashemi: wrote the manuscript, revision, correspondence. Aya Meslmany: did the technical work preparing to the surgery, critical revision. Bakr Haddad: managed the patient and did the surgery, the supervisor, patient care, revising critically. All authors read and approved the final manuscript.

## Patient perspective

The patients participated in the treatment decision and they were satisfied with the results of the treatment. Their perspective on this treatment was to regain the normal functions with good cosmetic outcomes.

## Consent

Written informed consent was obtained from the patient for publication of this case report and accompanying images. A copy of the written consent is available for review by the Editor-in-Chief of this journal on request.

## Research registration

No need.

## Guarantor

Dr. Bakr Haddad.

## Provenance and peer review

Not commissioned, externally peer-reviewed.

## Declaration of competing interest

The authors declare that there is no conflict of interest.
