# Supplementary material for: Facial defects reconstruction by titanium mesh bending using 3D printing technology: A report of two cases
Source: Ann Med Surg (Lond). 2022 May 20;78:103837. doi: 10.1016/j.amsu.2022.103837 (PMC9207074; doi:10.1016/j.amsu.2022.103837)
Supplement: Multimedia component 1 [file mmc1.docx]

| **SCARE 2020 Checklist** | | | |
| --- | --- | --- | --- |
| **Topic** | **Item** | **Checklist Item Description** | **Page Number** |
| **Title** | **1** | - Facial Defects Reconstruction by Titanium Mesh Bending using 3D Printing Technology: A report of two cases | 1 |
| **Key Words** | **2** | - Facial defect, titanium mesh, war injury, polylactic acid, case report. | 1 |
| **Abstract** | **3a** | - They were treated using preshaped 3D titanium mesh implants that were made using polylactic acid (PLA) material. - It has achieved a better outcome in comparison with manual bent titanium mesh in terms of anatomical symmetry, overall operating time, functional and esthetic impairment. | 1 |
|  | **3b** | - This study was conducted on two patients with severe war injuries; | 1 |
|  | **3c** | - they both suffered from a significant loss in one or more of the following bones: the zygomatic bone, maxilla, nasal bone, infraorbital rim, and mandible. | 1 |
|  | **3d** | - They were treated using preshaped 3D titanium mesh implants that were made using polylactic acid (PLA) material. | 1 |
|  | **3e** | - These points helped achieve better care for both civilian and war injuries associated with bone loss. | 1 |
| **Introduction** | **4** | - The most commonly affected bones in facial trauma are orbital walls and floor, which may lead to several complications such as diplopia, visual acuity disturbance, enophthalmos, and hypogeous Rationale - In the last 45 years, alloplastic methods have been used for tiny damages; in addition, they have a lot of benefits, for example, tinny, bio-compatibility, solid and bright, radio-opaque without creating artifacts in radiographic investigations. - This case was prospectively reported two patients suffered from a war injury that caused significant bone loss in the face in Aleppo, Syria, and managed in a low-expensive procedure. | 1 |
| **Patient Information** | **5a** | - A 19-year-old male was referred to our institution in 2021. - The other case was an 18-year-old female. | **2** |
|  | **5b** | - A 19-year-old male was referred to our institution in 2021. - The other case was an 18-year-old female who was referred to our institution for evaluation and treatment plan 8 years after a war injury. | 2 |
|  | **5c** | Past Medical and Surgical History   - Include any previous interventions and relevant outcomes. | N/A |
|  | **5d** | Drug History and Allergies   - Specify any acute, repeat, and discontinued medications. - Include any allergies and/or adverse reactions. | N/A |
|  | **5e** | Family History   - Health information regarding first-degree relatives, specifying any inheritable conditions.   Social History   - Indicate smoking, alcohol, and recreational drug use. - Level of social independence, driving status, and type of accommodation.   Review of Systems   - If appropriate, report on any other information gathered outside of the focused history. | N/A |
| **Clinical Findings** | **6** | - He suffered from a severe gunshot wound injury (GSW) that caused significant loss in the zygomatic bone, maxilla, nasal bone, infraorbital rim, and mandible with extensive wounds in the skin and the soft tissue. | 2 |
| **Timeline** | **7** | - institution for evaluation and treatment plan 8 years after a war injury to the left side of the face, including left eye loss. | 2 |
| **Diagnostic Assessment and Interpretation** | **8a** | - he underwent computed tomography (CT) scan to generate multislice 3D modeling of both affected and unaffected face bones. - She has also undergone a CT scan to generate slices for the 3D modeling template. | 2 |
|  | **8b** | Diagnostic Challenges   - Where applicable, describe what was challenging about the diagnoses (e.g. access, financial, cultural). | N/A |
|  | **8c** | Diagnostic Reasoning   - Describe the differential diagnoses, why they were considered, and why they were excluded. | N/A |
|  | **8d** | Prognostic Characteristics   - Include where applicable (e.g. tumour staging). | N/A |
| **Intervention** | **9a** | Pre-Operative Patient Optimisation   - Lifestyle (e.g. weight loss). - Medical (e.g. medication review, treating any relevant pre-existing medical concerns). - Procedural (e.g. nil by mouth, enema). - Other (e.g. psychological support). | N/A |
|  | **9b** | - The intervention on the area was using the Weber Ferguson surgical approach, which includes an incision under the lower eyelid extending to an incision on the side of the nose, then an incision around the nasal wing, finally an incision on the middle line of the upper lip. Then, after sterilizing the mesh, the patient's specific manufactured titanium was implanted and fixed using mini-screws made of titanium as well | 2 |
|  | **9c** | - Regarding both cases, the software package processed the CT slices to provide a mirrored 3D shape of the skull, identical to that of the injured young man, including the affected and the unaffected bones. Our simple 3D printer (Ender 3 Pro 3D printer, China) uses polylactic acid (PLA) materials for producing 3D printed objects which have excellent biocompatibility. | 2 |
|  | **9d** | Operator Details and Setting of Intervention   - Where applicable, include operator experience and position on the learning curve, prior relevant training, and specialisation (e.g. ‘junior trainee with 3 years of surgical specialty training’). - Specify the setting in which the intervention was performed (e.g. district general hospital, major trauma centre). | N/A |
|  | **9e** | Deviation from Initial Management Plan   - State if there were any changes in the planned intervention(s), and describe these alongside the rationale (e.g. delays to intervention). | N/A |
| **Follow-Up and**  **Outcomes** | **10a** | Specify Details regarding the Follow-Up   - When (e.g. how long after discharge, frequency, maximum follow-up length at time of submission). - Where (e.g. home via video consultation, primary care, secondary care). - How (e.g. telephone consultation, clinical examination, blood tests, imaging). - Any specific long-term surveillance requirements (e.g. imaging surveillance of endovascular aneurysm repair or clinical exam/ultrasound of regional lymph nodes for skin cancer). - Any specific post-operative instructions (e.g. post-operative medications, targeted physiotherapy, psychological therapy). | There was no follow-up |
|  | **10b** | Intervention Adherence and Compliance   - Where relevant, detail how well the patient adhered to and tolerated the advice provided (e.g. avoiding heavy lifting for abdominal surgery, or tolerance of chemotherapy and pharmacological agents). - Explain how adherence and tolerance were measured. | N/A |
|  | **10c** | Outcomes   - Expected versus attained clinical outcome as assessed by the clinician. Reference literature used to inform expected outcomes. - When appropriate, include patient-reported measures (e.g. questionnaires including quality-of-life scales). | N/A |
|  | **10d** | Complications and Adverse Events   - Precautionary measures taken to prevent complications (e.g. antibiotic or venous thromboembolism prophylaxis). - All complications and adverse or unanticipated events should be described in detail and ideally categorised in accordance with the Clavien-Dindo Classification (e.g. blood loss, length of operative time, wound complications, re-exploration or revision surgery). - If relevant, was the complication reported to the relevant national agency or pharmaceutical company. - Specify the duration of time between completion of the intervention and discharge, and whether this was within the expected timeframe (if not, why not). - Where applicable, the 30-day post-operative and long-term morbidity/mortality may need to be specified. - State if there were no complications or adverse outcomes. | None |
| **Discussion** | **11a** | - The use of digital surgical technologies has developed in CM reconstructive surgeries, as they enhance surgical procedures and lead to better outcomes for restoring both form and function. - The combination between three-dimensional (3D) techniques and radiology allowed the surgeon to better understand the anatomical surgical location of the patient and its exact pathology because every patient is a unique case that requires its own understanding. | 3 |
|  | **11b** | - Due to the unavailability of the titanium powder in wartime and the expensive tools of this approach, we formed the implants preoperatively on a 3D skull model using a simple 3D printer. | 3-4 |
|  | **11c** | - Raisia et al. found in their study that using custom-made implants in the reconstruction of orbital floor fracture led to better results when compared to intraoperative manual bending. - Similar studies reported the reconstruction of maxillary and mandibular deformities using a custom-made titanium mesh. - Cui et al. discussed in their study the application of pre-shaped titanium implants on a 3D skull model in the CMF reconstructive surgery. | 3-4 |
|  | **11d** | - The three-dimensional printing technique, using polylactic acid (PLA) for preparing titanium mesh, has shown better outcomes in restoring the bone structure and maintaining function. | 4 |
|  | **11e** | - As a result of this approach, facial symmetry was achieved using advanced digital technologies but also cost-effective materials. Pre-shaped mesh plated shortened the surgical duration due to complete implant preparation preoperatively and did not require great adjustments in comparison with the intraoperative approach. Postoperative trauma was reduced in comparison with the conventional approach. | 4 |
| **Patient Perspective** | **12** | - The patients participated in the treatment decision and they were satisfied with the results of the treatment. Their perspective on this treatment was to regain the normal functions with good cosmetic outcomes. | 4 |
| **Informed Consent** | **13** | - Written informed consent was obtained from the patient for publication of this case report and accompanying images. A copy of the written consent is available for review by the Editor-in-Chief of this journal on request. | 4 |
| **Additional Information** | **14** | - Writing case repots does not require ethical approval in University hospitals in our country. - The authors declare that there is no conflict of interest. | 4-5 |
| **Clinical Images and Videos** | **15** | - Fig. (1) 3D reconstruction based on CT scan slices in the first case - Fig. (2) Molding titanium mesh according to 3D printed template in the first case. - Fig. (3) Postoperative aspect showing the reconstructed area in the first case | 2 |
| **Referencing the Checklist** | **16** | - This case report has been reported in line with the SCARE 2020 Criteria [5]. | 1 |
